# Commercial ultrasounds: Blurring and renegotiating boundaries in prenatal care

**DOI:** 10.1177/13634593251394740

**Published:** 2025-11-17

**Authors:** Marjaana Jones

**Affiliations:** 1 Tampere University, Finland

**Keywords:** boundary work, commercialization, maternity care, prenatal ultrasounds

## Abstract

Prenatal ultrasounds have become a routine component of pregnancy care and a significant social milestone for expectant parents. Although originally developed for medical diagnostics, these technologies also carry important social and emotional connotations. This study examines the growing phenomenon of commercial prenatal ultrasounds in Finland, focusing on how these services blur traditional boundaries in maternity care. The research draws on data from private providers’ websites, interviews with healthcare professionals who perform commercial ultrasounds and with new parents who opted for additional scans during pregnancy. Using reflexive thematic analysis and the concept of boundary work, the study identifies three key areas of contestation: professional remits, the clinical versus non-clinical nature of ultrasound services, and the timing and autonomy of care decisions. The findings suggest that commercial ultrasounds challenge established professional hierarchies, reshape parental agency, and contribute to the commodification of pregnancy. The study highlights the need for further research into the emotional dimensions of prenatal care and the implications of expanding private maternity services in publicly funded healthcare systems.

## Background

Obstetric ultrasonography, or prenatal ultrasound, is a commonly used technology that has become a routine part of pregnancy care in several countries (Ibrahimi and Mumtaz, 2024; Whitworth et al., 2015). From a medical perspective, prenatal ultrasounds provide a way to estimate gestational age and the amount of amniotic fluid, monitor fetal health, detect multiple pregnancies, and diagnose a wide range of medical conditions (American Academy of Pediatrics, 2021). A certain number of scans may be offered to expectant parents for medical reasons as part of routine maternity care. These clinical ultrasounds are often traditional 2D scans. However, as this technology has developed and become more widely available, commercial providers have also begun to offer pregnancy ultrasound scans, which expectant parents are able to purchase (Metselaar, 2022; Roberts et al., 2015; Thomas, 2017). Commercial ultrasounds are often three-dimensional scans, which facilitate the detection of certain conditions and produce appealing images of the child-to-be’s face, or even 4D scans, which provide a “movie” of the fetus (Favaretto and Rost, 2025; Roberts, 2016).

In this study, prenatal ultrasounds are explored from a sociocultural perspective that focuses on the complex meanings, expectations, and tensions related to the use of this technology. The focus is specifically on commercial ultrasounds, as their increasing availability is a new development in Finland. They also connect Finland to a broader trend in which private clinics advertise additional *boutique ultrasounds* (Denbow, 2019), *bonding scans* (Roberts, 2016), or *entertainment ultrasounds* (Metselaar, 2022) directly to expectant parents. There are also indications that the availability of commercial ultrasounds is creating new practices or rituals around pregnancy, including the normalization of attending “extra” scans (Halle et al., 2018).

The aim of this study is to contribute to the literature on the commodification of pregnancy and the shifting relationships between expectant parents and the professional groups providing pregnancy care. Commercial ultrasounds provide a fruitful site for exploration. They have the potential to challenge traditional medical authority by offering services without clinical justification and to promote consumer autonomy, allowing parents to choose how and when to visualize the fetus (Denbow, 2019). The widening availability of commercial ultrasound scans has also faced criticism. The availability and marketing of new technology such as four-dimensional scans has transformed the prenatal clinic into a site of consumption (Thomas and Lupton, 2016) and led to a situation where professionals use medical technology for “entertainment” purposes. In the Finnish context, commercial ultrasounds challenge the long-established status quo of Finnish maternity care, potentially leading to various tensions and boundary negotiations as ultrasound moves into private commercial spaces.

I will begin by summarizing some of the previous literature on the varied meanings and uses of prenatal ultrasounds. Following this, I will provide contextual information regarding the site of this study and introduce the concept of boundary work, which I use to explore commercial ultrasounds in contemporary prenatal care.

### Medicalization and agency

Prenatal ultrasound technology has received a great deal of critical attention. It has been argued that making ultrasounds a routine part of maternity care has contributed to the medicalization of pregnancy and shifted the focus from natural processes to clinical surveillance (Favaretto and Rost, 2025; Rothman, 2014). These developments have allowed normal pregnancy to be defined as a risky condition and pregnant bodies to be configured as “at-risk” (Lupton, 2012; Thomas and Lupton, 2016). The routine use of ultrasound technology can also compel women to make complex decisions about the pregnancy and the fetus based on probabilities and risk calculations (Moncrieff et al., 2021). Ultrasound technology and the images it produces have also been used by politically motivated groups, who have deployed ultrasounds to help construct the fetus as an individual person, separate from the pregnant woman (Denbow, 2019).

As ultrasounds have become part of routine care in several countries, the expectation that women should participate in this form of self-surveillance during pregnancy has intensified (Thomas, 2017). Indeed, they have become so enshrined that some studies have questioned whether expectant parents truly have autonomy or choice in attending them, or whether they do so routinely following authoritative advice from health professionals to ensure the wellbeing of the baby (Åhman et al., 2019). Ultrasound scans have become intertwined with moral expectations of responsible parenthood, and by attending them, expectant parents—particularly pregnant women—can demonstrate care for their unborn child (Daniels, 2023). Hence, it can be argued that this practice reinforces gendered expectations of maternal responsibility and emotional labor (Denbow, 2019). It has also been suggested that interactions during the scan can reduce parental agency. If parents are not informed about what is happening during the scan, professionals may reinforce a sense of passivity and dependence on medical authority (Yeh, 2023).

### Identity work and emotional ambivalence

Expectant parents engage in identity work during ultrasound as they actively interpret and negotiate their roles as mothers, fathers, patients, and consumers of medical technology (Yeh, 2023). Indeed, the scan room provides an arena in which expectant parents, and to a lesser extent grandparents and siblings, rehearse their new identities (Åhman et al., 2019; Skelton et al., 2024). Choosing who to take to the ultrasound appointments and who to show pictures to matters, as these actions can also signal who will be part of the new baby’s family (Roberts et al., 2015). Ultrasound scans can also produce feelings of awe and pride and increase feelings of attachment for grandparents (Roberts et al., 2017). Hence, ultrasound images are not just diagnostic tools—they are also emotionally and socially meaningful as expectant parents use them to imagine and relate to their unborn child, reinforcing their parental identity (Yeh, 2023). Additionally, seeing the fetus can contribute to imagining the baby as a real person and strengthening emotional bonds (Skelton et al., 2024). Partially due to these emotional and social factors, attending an ultrasound during pregnancy has become an anticipated social landmark (Skelton et al., 2024).

Studies focusing specifically on commercial ultrasounds suggest that they can act as an additional opportunity to strengthen family relationships (Roberts et al., 2015, 2017). Denbow (2019) has argued that commercial providers also market their ultrasound services by emphasizing the bonding aspects, visualization and emotional experiences over medical diagnostics. Additionally, the services are often framed around love and family connection, using consumer-friendly language and imagery.

Research that has explored ultrasounds from a parental perspective, highlights their use for reassurance (Roberts et al., 2015; Thomas, 2017; Westerneng et al., 2019). Indeed, engagement with medical technologies during pregnancy can address uncertainties, alleviate anxieties and shape the experience of pregnancy more broadly (Ross, 2018). Ultrasound attendance can also be conceptualized as a ritualized practice that reassures both healthcare providers and expectant parents by symbolically demonstrating that care is being taken—even when no intervention follows (Daniels, 2023).

Nevertheless, although some gain reassurance from prenatal ultrasounds, they can also have the opposite effect and lead to harmful consequences, including anxiety and adverse psychological effects for pregnant people and their partners (Ahman et al., 2010; Curado and Bhide, 2018). These feelings can be shaped by several factors such as the clinical environment, the sonographer’s communication style, and the broader context of healthcare inequality (Daniels, 2023). A study by Favaretto and Rost (2025) has highlighted the power of ultrasounds as visual technology, which also holds psychological risks. While many experienced joy during ultrasound, unexpected or unclear findings could cause distress, influence reproductive decisions, and lead to long-term psychological impacts—even when outcomes were ultimately positive (Moncrieff et al., 2021).

### Professional and ethical challenges

Studies focusing on health professionals’ perspectives on prenatal ultrasounds further emphasize the idea that ultrasounds have become an unquestioned and normalized part of pregnancy care (Edvardsson et al., 2015a). This has led to concerns that parents may place too much trust and reliance on ultrasounds and may be unaware of the limitations of the technology and its potential disadvantages, such as false positive and false negative findings (Edvardsson et al., 2015b).

Concurrently, professionals might find it challenging to communicate complex information to expectant parents and may have difficulties in translating anatomical images into clear, meaningful information about the fetus, while also providing adequate counseling (Edvardsson et al., 2015b; Stephenson et al., 2017). Indeed, providers have expressed concerns about diagnostic errors and emphasized the need for adequate training and sufficient time with patients (Moncrieff et al., 2021).

Health professionals can also face ethical dilemmas, as there may not be clear guidance on which markers or findings should be disclosed (Edvardsson et al., 2016). Moncrieff et al. (2021) have also drawn attention to the attitudes and communication styles of health professionals conducting the scans, as these can significantly affect the parental experience. In relation to commercial ultrasounds, concerns have been raised about the lack of professional standards and the operation of services in a legal gray area, often outside the oversight of medical regulation (Denbow, 2019).

## Clinical and commercial ultrasounds in Finnish maternity services

The data for this study was collected in Finland, which has a long-established maternity care pathway, comprising of community-based clinics [äitiysneuvola] run by multiple public health nurses and one or two general physicians (Schmidt and Bachmann, 2021). These tax-funded services continue to reach most expectant parents (Tiitinen, 2023a). The clinics provide regular check-ups throughout pregnancy and can refer to other specialist services. The first meeting with a public health nurse is usually offered between weeks 8 and 12 and the clinics provide regular check-ups approximately once a month. The public service also includes two prenatal ultrasound appointments, which are usually performed either by a sonographer or a midwife, or in some cases a specialist physician at a local hospital. These ultrasound appointments are not compulsory, but they are attended by 95% of expectant parents (Tiitinen, 2023a). The early pregnancy ultrasound usually takes place during weeks 10–13 of pregnancy. The second ultrasound examination for severe structural conditions is conducted during weeks 18–21 or after week 24 + 0 of pregnancy (Tiitinen, 2023b). In this study, these will be referred to as clinical ultrasounds.

Despite the comprehensive availability of tax-funded public maternity services in Finland, an increasing number of expectant parents are turning to the private health sector to supplement their care. Over the past decade, a growing range of commercial providers—from large healthcare corporations to small midwife-led businesses —have entered the maternity care market, offering services that often mirror those available in public clinics. This trend signals a shift in consumer behavior and raises questions about the adequacy and appeal of public services. This study focuses on one particularly visible segment of the private market: commercial or “entertainment” ultrasounds. These services, provided by doctors, midwives, or ultrasound technicians, are easily accessible through online or phone booking. However, unlike public services, they are not subsidized by the state and require out-of-pocket payments ranging from 90 to 300 euros, making them financially inaccessible to some. The emergence and popularity of such services suggest that public maternity care is no longer the sole or default option, but rather one among several competing choices in a diversifying landscape. In this study, I will apply the concept of boundary work to explore the tensions and shifts commercial ultrasounds are creating in this Nordic context. This study will also shed further light on the reasons behind the increase in commercial maternity services.

## Boundary work

This study uses the concept of boundary work to explore the negotiations and tensions related to commercial ultrasounds. Boundary work describes the ways individuals and groups establish, maintain, or challenge distinctions between social categories, professions, or knowledge domains. The concept was initially introduced to refer to ways in which science can be distinguished from non-science (Gieryn, 1983). It’s been described as “rhetorical games of inclusion and exclusion’, which are a step ‘toward a cultural interpretation of historically changing allocations of power, authority, control, credibility, expertise, prestige, and material resources among groups and occupations” (Gieryn, 1995:406). The concept has been applied in various ways in studies focusing on health and relationships between health professionals (e.g. Bucher et al., 2016; Cregård, 2018; Rapp et al., 2021). Boundary work can provide an insight into ways in which different professional groups, such as midwives, nurses and doctors, demarcate, change, or maintain their boundaries between groups, professions and organizations (Langley et al., 2019) or how patients are able to influence healthcare agenda-setting (Schölvinck et al., 2020). Boundaries are not perceived as being fixed (Meier, 2015). Instead, they are flexible and shift due to social, cultural and technological changes, which lead to renegotiation of roles and authority. Boundary work can be competitive where boundaries function as barriers that promote separation, or collaborative, where they enable collaboration (Langley et al., 2019).

In this study, the concept of boundary work is applied to examine the shifting landscape of prenatal care, particularly through the lens of commercial ultrasound services. In the Finnish maternity care context, several institutional and professional boundaries have traditionally governed the provision of prenatal ultrasounds. Public maternity services also operate under legislation (Finlex, 2011), which regulates the number and timing of clinical ultrasounds. Also, as medical procedures, ultrasounds may only be conducted by licensed healthcare professionals. However, the emergence and growth of commercial ultrasound services—offered outside the public system—challenge some of these established boundaries. By applying the concept of boundary work, this study explores how the relationship between public and private healthcare providers is being redefined, and how expectant parents negotiate their roles and agency within this evolving care environment. Specifically, the analysis focuses on:

1. What are the sites of boundary negotiations related to the provision and use of commercial ultrasounds?
2. Within these sites, how are power dynamics, roles, and responsibilities negotiated and reshaped between healthcare providers and expectant parents?

## Materials and methods

The materials were collected by as part of a research project focusing on the Finnish maternity care system and the commodification of pregnancy care titled “Futures of Finnish Maternity Care: Commercial, Political and Experiential Framings.” The project received approval from the relevant ethics committee. For the overall project, I conducted 20 interviews with private providers and 22 interviews with parents. For this study, I have chosen materials that directly address the provision and use of private prenatal ultrasounds. Hence, the materials selected for this study consist of interviews conducted with private providers offering commercial ultrasounds (*n* = 10), and interviews with new parents (*n* = 11) who purchased commercial ultrasounds during pregnancy. Additionally, I have included the websites of private providers (*n* = 9) offering commercial ultrasounds to gain a wider understanding of how private providers describe and discuss commercial ultrasounds.

I began the data collection in December 2022 by searching for companies offering pregnancy services using a search engine and the terms “private maternity clinic,” “private antenatal clinic,” “pregnancy monitoring,” and “private midwife.” These searches identified midwife- and nurse-led businesses, as well as medical centers currently offering pregnancy-related services. Service providers were included if they: 1) had their own websites, and 2) stated on their websites that they offer antenatal services or other pregnancy monitoring and/or childbirth preparation. Individuals listed as homebirth midwives without their own websites, as well as companies whose services focused on the postnatal period, were excluded. For the purposes of this study, I selected providers that advertise prenatal ultrasound services on their websites. From these providers’ websites, I extracted the parts of the texts that describe the ultrasound services.

The interviews with health professionals offering pregnancy ultrasound services were conducted between February and August 2023. I contacted all the private providers identified during the website search directly via email and invited them to participate in an interview. I approached small and midsize companies through their owners and applied for organizational permissions to contact professionals working for larger healthcare providers. All participants received written information about the project and were asked to provide both verbal and written consent prior to the interviews. The interviews were conducted either face-to-face or online, recorded, and transcribed verbatim. All interview data were stored securely, and any identifying information was removed during transcription to ensure participant anonymity.

For the professional interviews, I developed a semi-structured interview guide based on the research aims of the overall project. Each interview covered the following themes: professional background and personal experiences working in maternity services; perceptions of expectant parents’ needs and wishes; and services provided. However, as the professionals were working in different environments and provided different types of services, space was created for them to discuss issues they deemed relevant to their work. For this study, I have included interviews with professionals (doctors, midwives, and sonographers) who offer commercial ultrasound services. In the data analysis, I have included the parts of the interviews where they discuss prenatal ultrasounds.

In 2024, I conducted interviews with parents who had had a child within the last 2 years. I recruited them through interview calls published online on the project website and social media channels. These channels included groups aimed specifically at families with young children, which post activities in specific cities or regions. I also distributed information leaflets distributed to voluntary sector organizations offering activities for new parents. All interested parents then contacted me and were provided with written and verbal information about the project. All participants provided written consent prior to the interviews. The interviews were conducted online via Zoom and all participants were given the option to take part in either a dyadic or an individual interview. I developed and used a semi-structured interview guide based on the research aims of the overall project. The guide covers a range of themes, including family planning, pregnancy, childbirth, and service use experiences. All interview data were handled confidentially, and identifying details were removed during transcription to protect participant anonymity. For this study, I chose to analyze the interviews of parents who had used commercial ultrasound services during their pregnancy. These participants all had 1–2 children, lived in the Southern and Western parts of the country and were aged between 26 and 42.

The analysis was guided by Braun and Clarke’s reflexive thematic analysis (2021), which provided a flexible framework for identifying and interpreting patterns across the data. As part of this process, I used the concept of boundary work as an analytical tool to inform both the coding and the construction of themes. I began by reviewing the entire dataset to identify segments where prenatal ultrasounds were discussed. From there, I inductively coded the parts of the data that focused specifically on commercial ultrasound services. This stage aimed to uncover points of tension and moments of negotiation around issues such as credibility, responsibility, and expertise. The resulting codes were grouped to construct the main sites or issues of tension. Once this thematic structure was established, I continued to apply the concept of boundary work to examine more closely who was involved in these negotiations and how roles and responsibilities were attributed to different actors in discussions about commercial ultrasounds. Whilst refining the themes, I also compared and contrasted the findings with previous research on ultrasound practices.

I approached this study as a health researcher with a background in examining services from the service user perspective. While my previous professional experience in healthcare informs my broader understanding of care systems, I do not have direct experience of working within maternity care. Throughout the research process, I critically examined my own assumptions, values, and personal knowledge about pregnancy care, allowing me to remain open to diverse interpretations and experiences.

The final themes reflect my interpretive engagement with the data and are shaped by both the research context and my analytical lens. The names of the themes reflect the main sites of tension identified from the data and the content of each theme further elaborates on how different roles, responsibilities and power dynamics are being negotiated and reshaped. The first theme explores boundary negotiations between different professional groups, focusing on questions of authority and legitimacy in conducting prenatal ultrasounds. The second theme examines the dual clinical and non-clinical nature of commercial ultrasound services, highlighting how these services are positioned and understood by different actors. The third theme addresses negotiations around the timing of ultrasounds and the competing views on what constitutes appropriate or “correct” timing. In the following sections, I present the themes in more detail, focusing on the boundaries being negotiated and the shifting dynamics that emerge through the use and provision of commercial ultrasounds.

## Results

### Renegotiating professional remits

The provision of commercial ultrasounds directly engages with a decades-long discussion about professional responsibilities in Finnish maternity care. Midwives remain a central professional group responsible for childbirth in Finland. However, their ability to participate in pregnancy care has been limited since the 1970s (Finlex, 1972). While they can work in public maternity clinics, they must obtain an additional public health nursing degree to do so. This requirement has caused inter-professional tensions for decades, as the organization representing midwives has consistently asserted that they are the professional group best equipped to support expectant parents during pregnancy and prepare them for birth (Oinonen, 2022). For many midwives, operating private pregnancy services, owning an ultrasound machine, and offering commercial ultrasounds represent a way to renegotiate the division of labor in pregnancy care and expand their professional remit. Some interviewees even described this as a rebellious act. In the extract below, one interview participant describes the experience of acquiring an ultrasound machine:

*When I started thinking about this, there was a midwife-led service in* [another city]. *They were probably one of the first midwives who had bought themselves an ultrasound machine and started doing them. I though “wow, th’,s anarchy”, taking back the professional skills so it’s not just done working under a doctor. I thought, damn, I want one of those.* (Private midwife)

However, the act of breaking free from established professional hierarchies and offering commercial ultrasounds was not well received by everyone. This became evident when midwives working in the private sector described their collaboration and relationship with public services. In certain areas of the country, they felt they were met with hostility and that their professionalism was being questioned. These difficulties arose particularly in situations where an ultrasound scan led the midwife to suspect that something was not progressing as it should with the pregnancy, prompting a referral to the local hospital for further examination. Some felt that these referrals were dismissed, questioned, or criticized—particularly by doctors working in the hospitals.:

*I feel like crying when I talk about it* [the relationship with the local hospital]. *It’s really sad. Why does it have to be like this? I believe that I know what I’m doing and I’m good at this* [. . .] *I try to be constructive, but I feel that they criticise and evaluate me and question how people benefit from this* [service]. *Every time I discover a miscarriage during an appointment, I get anxious about referring the person to the* [hospital]. (Private midwife)

In the above extract, the private midwife shares their experiences of collaborating with the local hospital. In the data, commercial ultrasounds were referred to by some professionals as a *grey area* and a potential challenge to established professional hierarchies. In other words, they forced professionals to negotiate who oversees care and who administers tests and examinations during pregnancy. Commercial ultrasounds and subsequent referrals could be seen as an intrusion of the private sector into the established realm of public maternity care.

Some private midwives chose to collaborate with a doctor who could make referrals to the hospital on their behalf, thereby providing them with greater legitimacy in the eyes of professionals working at the local hospital. However, professionals offering commercial ultrasounds in the private sector did not automatically face these tensions. Those working for larger private healthcare companies seldom mentioned any difficulties when making referrals to the public sector.

Through their websites and during the interview discussions, professionals also made comparisons and expressed value judgments regarding different ultrasound providers. Commercial ultrasounds conducted by midwives were described as more holistic experiences that offered expectant parents an opportunity to discuss their hopes and fears. In contrast, some felt that such opportunities were not guaranteed during routine clinical ultrasounds. Additionally, midwives conducting commercial ultrasounds emphasized the financial aspects of these procedures. Based on information found on company websites, private doctors often charged 200–300 euros for an ultrasound appointment, whereas midwives’ pricing was roughly half that. Hence, private midwives were able to position themselves as providers of a more accessible and affordable service.

Despite these tensions and the desire to reconfigure the professional mix involved in pregnancy care, certain boundaries continued to be enforced in relation to ultrasounds—particularly regarding responsibilities for diagnostics. Even when midwives conducted the ultrasound examination, they did not provide a diagnosis. If further examination was needed, they referred the expectant parent to a doctor or the local hospital. This was also clearly conveyed to potential customers through the websites, which explained that::

*Midwives do not conduct structural exams on the fetus or perform screening ultrasounds. If you want a more detailed diagnosis of the baby’s health and the placenta’s function, it is advisable to book an appointment with a doctor instead of a midwife.* (Private providers website)

It is also important to note that professionals providing commercial ultrasounds were not the only ones participating in the negotiation of professional boundaries. For expectant parents, this was equally a process of boundary negotiation—deciding which professional groups they wanted to involve in their care during pregnancy. The decision to purchase an additional ultrasound could also represent a stance aimed at altering the current division of labor in prenatal care.

### Blurring clinical and non-clinical boundaries

*You can come to the ultrasound just to see and admire your future family member, listen to the heartbeat, or guess the baby’s gender.* (Private providers website)

The above quote is from the website of a private provider advertising commercial ultrasounds. Overall, the descriptions of commercial ultrasounds often combine medical language and reasoning with social and emotional aspects. On their websites, private providers actively encourage ultrasound appointments at any stage of pregnancy. The idea of using ultrasounds as a fun, bonding experience, is further reinforced by offerings such as 3D and 4D ultrasounds or memory sticks filled with pictures and videos. Commercial ultrasounds were also given names like “first date” or “see you soon.” This intentional use of wordplay and emotionally charged language further highlights the non-medical aspects of the service.

During the interviews, professionals also discussed the bonding and entertainment aspects of ultrasound scans. Unlike the routine clinical scans, these appointments were open to a much wider selection of family members and friends. The appointment room could be transformed into a space for bonding and sharing. In some cases, the ultrasound scan was incorporated into a larger family outing that included various shared rituals aimed at preparing everyone for the birth of a new family member::

Private midwife: *Some people have their sister of gran with them or even more people. We just pull up some more chairs and have the whole family or the baby’s godparents looking at the 3D ultrasound.*

Interviewer: *It sounds like an experience for the whole group.*

Private midwife: *Oh yes.*

Interviewer: *Some* [in earlier interviews] *have said that people visit Ikea and then. . .*

Private midwife: *Yes, exactly. Ikea or some other place. Especially when people travel from the countryside to the city. There’s a secondhand children’s clothing store and other touristy places to visit if you bring children along. And then they go back home. Once there was a gran who came to see the baby with her granddaughter. She was about 80 and had never had an ultrasound scan and she was telling about her own pregnancies and deliveries and I was like “wow, where else could you hear these stori”,. These are wonderful moments for them.*

However, trying to provide a joyful experience for the whole family could also come with certain drawbacks. Ultrasound scans could reveal sad or difficult information, which could be challenging to discuss with older children or with a large group of loved ones in the room.

Based on the data, professionals were highly aware of the social and emotional significance of ultrasound scans and participated in the blurring of medical and non-medical boundaries. Thomas (2017) has argued that professionals who engage in this must carefully negotiate a balance between “offering expertise and medically based reassurance with providing a joyful experience for parents as consumers” (p. 359). Turning ultrasounds into commodities also came with a set of challenges. Professionals attempted to strike a balance by providing medical information and guidance alongside the light and amusing descriptions found on websites. They also emphasized their knowledge, clinical expertise, and training to reassure that their work was not done merely for entertainment purposes. As the extract below shows, commercial ultrasounds—and particularly their use for non-clinical purposes—provoked conflicting feelings among professionals, with some of the interviewees questioning their widening availability and use::

*I do think that, for most people, extra ultrasounds aren’t necessarily something they truly need—unless there are real challenges in bonding with the baby. I do recognize that in such cases, they can be beneficial and certainly not a waste of money. However, I also feel that bonding could be fostered in other ways as well.* (Private midwife)

Additionally, professionals highlighted that, even though ultrasounds could be purchased for a variety of reasons, they continued to serve a medical purpose. In the extract below, the professional draws a clear boundary—emphasizing that commercial ultrasounds also serve a medical function and reinforcing their role as a knowledgeable expert::

*I need to explain to my colleagues and the families that attend my service that I do things first and foremost from a medical perspective. These are not entertainment ultrasounds. The* [families] *attend 3D ultras and hope to get perfect 3D images. I tell them that images depend on the baby’s position and our ultrasound machine, the position of the baby, the amount of amniotic fluid and the placement of the placenta. They are not experts on this and it’s understandable that they expect me to just press a button and produce perfect 3D images. So I need to set things straight.* (Private midwife)

The above quote further highlights the professional ambivalence toward commercial ultrasounds. While professionals wanted to offer families opportunities to bond, they also remained concerned about using ultrasounds for entirely non-clinical reasons.

Expectant parents also had their own perspectives on the boundary between clinical and non-clinical use. Interestingly, the parents interviewed for this study did not emphasize the entertainment aspects of commercial ultrasounds. Instead, clinical reasons dominated their explanations for attending these scans. Expectant parents wanted to ensure that the fetus was growing and developing normally, and that the pregnancy was progressing as expected.

Later in pregnancy, commercial ultrasounds were sometimes used to determine the baby’s gender, but for many interview participants, the scans were primarily a way to prepare for delivery, for example, by checking the baby’s measurements. Another key motivator was emotional reassurance, which could be gained both from seeing the fetus on the monitor and from the professional conducting the scan.

### Negotiating timing and choice

Commercial ultrasounds shape the relationship between expectant parents and health professionals working in both the public and private sectors. Private providers have made commercial ultrasound scans available throughout pregnancy. As a result, expectant parents with enough disposable income to pay for additional services, are no longer required to wait until the first routine clinical scan. Based on information gathered from private providers’ websites, expectant parents are able to book a commercial ultrasound as early as week six. Additionally, there are no limits on the number of ultrasound appointments they could potentially attend during pregnancy.

Parents have the opportunity to independently restructure their pregnancy pathway, and by doing so, they challenge the gatekeeper role that health professionals have traditionally held over the use of this technology. Expectant parents can assess and decide whether an ultrasound is needed, as well as when and where to have it. For those attending a commercial ultrasound scan in early pregnancy, the private provider also becomes their first point of contact by default—a role traditionally reserved for professionals working in public maternity clinics.

Reassurance has been a recurring theme in literature regarding the use of ultrasound technology, with several studies arguing that expectant parents attend ultrasound scans to gain assurance and combat anxiety (Roberts et al., 2015; Thomas, 2017; Westerneng et al., 2019). In the interviews conducted for this study, expectant parents’ desire to renegotiate the timing of ultrasounds often stemmed from previous experiences of miscarriage or other struggles during earlier pregnancies::

*I have had miscarriages before, so during the first trimester, it felt like Schrodinger’s baby. So, we had a private early pregnancy ultrasound and everything was fine. We tried to have a scan in the public sector before week 12 for the sake of my mental health, but I have to say that the doctor didn’t really understand why we wanted an additional scan, so we didn’t get one. Up until week 12 it was scary, but once I began to feel the baby move and I could count the movements, it started to get easier.* (Parent)

The choice to attend commercial ultrasound appointments suggests that, in some cases, the care offered by public providers is out of sync with parental needs. Commercial ultrasounds offered a way for parents to renegotiate the timing and rhythm of care. For the parents interviewed in this study, commercial ultrasound scans appeared to provide a form of relief, especially in early pregnancy. The lack of publicly available scans left some parents in limbo, forcing them to deal with their anxieties alone. As one interviewee expressed, waiting for the first routine scan “*just feels like an impossible thought*,” particularly in situations where an earlier pregnancy had ended in miscarriage.

For some, early pregnancy ultrasounds provided reassurance that the pregnancy was “*real*” or viable, allowing them to feel joy and begin bonding. They needed to see the heartbeat and ensure they were not attending maternity clinic appointments “*for nothing*.” Hence, commercial ultrasounds were used to protect parents from disappointment and heartbreak, as 10–12 weeks “*is such a long time to be in the belief that everything is fine*.”

Later in pregnancy, ultrasound scans could be booked to find out the baby’s gender or to ensure the pregnancy was progressing normally. There were also instances where parents chose to book additional scans because they did not trust the information given by health professionals or were unable to access specific medical checks. In the extract below, one parent had been concerned about the size of their baby::

*Well, I didn’t get a referral to get a fetal size estimate from the public sector, even though I had really wished for one. Then we went private. At that point, I had attended so many ultrasounds that we were low on money, but my mom agreed to pay for the ultrasound for me.* (Parent)

As mentioned in an earlier section, private providers often encourage the use of commercial ultrasounds whenever and as often as parents deem necessary. However, the extract above demonstrates one of the challenges associated with this newfound “freedom.” Due to out-of-pocket payments, commercial ultrasounds can become a financial burden for families trying to cope with the uncertainties of pregnancy.

Private providers also recalled situations where expectant parents attended several additional scans—sometimes almost on a weekly basis. Hence, the perceived freedom offered by commercial scans does not automatically lead to an increased sense of autonomy and agency, but rather to a state of dependence on this technology. This highlights how ultrasound scans can potentially generate more stress and how they may be used as a substitute by expectant parents trying to manage pregnancy-related anxieties.

## Discussion

In this study, I have examined the boundary negotiations surrounding commercial pregnancy ultrasounds, drawing on data collected in Finland. Three primary sites of negotiation emerged from the analysis: firstly, debates over who should be authorized to perform pregnancy ultrasounds; secondly, the ways in which the growing availability of commercial ultrasounds contributes to the blurring of boundaries between clinical and non-clinical practices; and thirdly, discussions about who determines the appropriate timing for offering and attending ultrasounds.

The growing interest in commercially available ultrasounds appears to be a trend not only in Finland but also across Western Europe and the Nordic countries (e.g. Halle et al., 2018; Löwy, 2022; Marker et al., 2023; Metselaar, 2022). These additional scans are particularly popular early in pregnancy, and studies have even suggested that they are becoming a normalized part of the pregnancy experience, creating new patterns of consumption and pregnancy culture (Halle et al., 2018; Jones and Lahtinen, 2025).

In Finland, the established status quo of maternity care involves services provided through maternity clinics and public hospitals. The increasing involvement of the private sector represents a new development that is likely to generate inter-sectoral tensions. According to the findings, these tensions arise especially in situations where private providers must refer patients to public hospitals for further testing or diagnostics.

However, the main source of tension may not be that midwives are conducting ultrasounds and making referrals —since they are also among the primary professionals performing them in hospitals—but rather that they are expanding their professional remit into pregnancy care and asserting greater autonomy. By independently offering prenatal care and services, midwives continue to renegotiate the midwife-doctor relationship, a longstanding point of contention in maternity services (Najmabadi et al., 2020; Watkins et al., 2025). Despite ongoing debates over professional boundaries, it was widely acknowledged that diagnostics remain clearly within the domain of medical professionals.

The growing availability of commercial ultrasounds and other medical services is also reshaping the role of healthcare professionals and contributing to the commodification of pregnancy and prenatal care. Traditionally, health professionals have acted as gatekeepers to medical technologies (Spalletta and Green, 2025). This role may be gradually diminishing as parents gain the ability to book ultrasounds directly and at their own discretion. The findings of this study further support the assertion that commercial ultrasounds are blurring the boundary between clinical and non-clinical practices (Denbow, 2019; Thomas, 2017). These scans are performed by trained professionals who collect clinical information about the fetus, yet private providers also market them as joyful experiences—opportunities to “say hello” to the baby. The keepsake images and videos enhance the experiential aspect of the practice, transforming the technology into a commodity that can be purchased by anyone at any time.

This study has further highlighted that commercial ultrasounds are being marketed and consumed as more than just clinical examinations. Indeed, this was portrayed as one of the main strengths of commercial ultrasounds by private providers. They claimed to consider the whole family unit and recognize the emotional and bonding aspects of the scan. According to them, this was something that routine clinical ultrasounds often failed to do. Prior literature on the use of commercial ultrasounds has similarly emphasized that the appointment itself, the image on the screen, and the photos or videos that parents can take home often provide a sense of reassurance (Roberts et al., 2015; Thomas, 2017; Westerneng et al., 2019). As commercial ultrasounds are entirely optional, they offer expectant parents new forms of active agency, allowing them to seek scans for emotional comfort (Metselaar, 2022).

However, referring to commercial ultrasounds merely as non-clinical or entertainment scans can obscure some of the motivations, ambivalence, and reservations associated with them. Although they were advertised as fun bonding experiences, parents primarily described using them either for clinical reassurance or to alleviate anxiety. Some of their accounts align with Rothman’s (1993) concept of *tentative pregnancy*, which refers to the emotional and psychological state of uncertainty that many experience during pregnancy when prenatal diagnostic technologies are involved. Indeed, several parents explained that they were holding back emotionally and not allowing themselves to feel happy or certain about the pregnancy until they had attended the ultrasound. Ross (2018) has written about a similar phenomenon in relation to home pregnancy tests, suggesting that a positive result made the pregnancy feel real and tangible. In this study, however, the pregnancy test played a minor role, as certainty was only achieved by seeing the fetus and hearing the heartbeat.

These findings underscore the central role that technologies like ultrasound play in shaping the modern pregnancy experience. Several studies have also highlighted that the medical information gained through these scans does not necessarily lead to parental empowerment (Daniels, 2023; Moncrieff et al., 2021; Rothman, 1993; ). While commercial ultrasounds offer the appearance of increased agency and peace of mind, they may also heighten anxieties (Ahman et al., 2010; Curado and Bhide, 2018) and can even become a financial burden on families.

In the later stages of pregnancy, commercial ultrasounds were used by parents as a means of preparing for birth. Some expectant parents felt that their concerns—such as those related to fetal size—were not taken seriously in public care settings, so they turned to private providers to gain reassurance from professionals they perceived as more attentive. Others sought commercial ultrasounds simply to feel cared for and acknowledged, especially in cases where they had experienced a previous miscarriage or other complications.

These findings raise broader questions about the availability of psychological support during pregnancy. A recent report suggests that there is significant regional variation in how well parents feel their emotional needs are being met (Klemetti et al., 2024). In such situations, commercial ultrasounds may be used by some as a substitute. Earlier research has highlighted the ethical and moral dilemmas posed by prenatal ultrasound technology (Åhman et al., 2019; Favaretto and Rost, 2025). These concerns also extend to private providers, some of whom expressed ambivalence about the increasing and unrestricted use of medical technology.

Overall, this study has explored the growing commercial use of ultrasound technology in a country that already offers relatively comprehensive public maternity services. Although this is a small-scale study highlighting the perspectives of those providing and purchasing these services, the use of multiple data sources has allowed me to uncover the varied tensions and negotiations taking place. In the future, it will be important to further investigate how the emotional needs of expectant parents can be met, so that these technologies do not become the sole means of managing anxiety. It is also worth examining the consequences of the ever-increasing availability of commercial services on parental expectations and pregnancy experiences. From a service and patient safety perspective, it is important to understand how responsibilities are negotiated and shared between public and private sector providers, as these can have direct consequences care received by expectant parents.
